# Laparoscopic Reversal of Roux-en-Y Gastric Bypass for Isolated Refractory Postoperative Nausea: A Case Report

**DOI:** 10.7759/cureus.106464

**Published:** 2026-04-05

**Authors:** Una Gadzane, Maksims Mukans, Arturs Flaksmanis, Igors Troickis

**Affiliations:** 1 Faculty of Medicine, Riga Stradiņš University, Riga, LVA; 2 Department of General Surgery, AIWA Clinic, Riga, LVA

**Keywords:** bariatric surgery, bariatric surgery complications, obesity, refractory nausea, reversal surgery, roux-en-y gastric bypass surgery

## Abstract

Reversal of Roux-en-Y gastric bypass (RYGB) is an uncommon but sometimes necessary intervention for patients who develop severe, therapy-resistant postoperative symptoms, for example, dumping syndrome, excessive weight loss, and refractory nausea.

We report the case of a 39-year-old female who developed persistent nausea, anorexia, weakness, and profound weight loss following uncomplicated RYGB. Despite nutritional counselling, antiemetic therapy, and subsequent laparoscopic repair of Petersen’s defect, her symptoms recurred without identifiable radiologic or biochemical abnormalities. Given the persistence and intensity of her symptoms, a laparoscopic reversal to normal anatomy was performed 15 months after the initial operation. Postoperatively, she experienced complete resolution of nausea and anorexia, with weight stabilisation and remission of prior musculoskeletal pain and gastroesophageal reflux disease, which was confirmed at the four-month follow-up.

This case highlights the diagnostic and therapeutic challenges associated with refractory postoperative nausea after RYGB, the need for careful multidisciplinary evaluation, including consideration of psychological contributors, and the role of reversal surgery as a last resort when conservative management fails. Bariatric surgery effectively treats obesity, but rare complications such as refractory nausea can increase morbidity, as illustrated by the present case. According to the current literature, management should prioritise conservative therapy, with surgical intervention reserved for unresponsive cases. This report has several limitations, including limited conservative management before reversal, an absence of an in-depth psychiatric evaluation, and a short postoperative follow-up period. To the best of the authors’ knowledge, this is the only reported case describing isolated, persistent nausea following RYGB in the absence of identifiable structural or biochemical abnormalities.

## Introduction

Obesity remains a prominent and growing global health challenge [1,2]. Projections indicate that the total number of adults living with obesity is set to increase by over 115% between 2010 and 2030, rising from 524 million to an estimated 1.13 billion individuals [2]. Bariatric surgery remains the most durable and effective treatment modality for severe obesity and its related comorbidities [2,3]. The Roux-en-Y gastric bypass (RYGB) is widely recognised as a highly effective intervention, with extensive evidence supporting sustained weight loss and significant improvements in comorbidities such as hypertension, diabetes mellitus, and hyperlipidaemia [4-6].

Despite these benefits, a small subset of patients undergoing RYGB experience complications that require additional intervention [3,5]. Many long-term issues, including marginal ulcers, dumping syndrome, and micronutrient deficiencies, can often be managed conservatively or with limited surgical revisions. However, a minority of patients develop severe and refractory symptoms that may necessitate a complete reversal of the RYGB to normal anatomy [1,7,8]. RYGB reversal is considered rare, with reported rates of approximately 0.2%-0.6% of all RYGB procedures [3].

Recent multicentre analyses indicate that the most common indications for RYGB reversal include dumping syndrome (33.3%), excessive weight loss or malnutrition (29.2%-53%), and refractory hypoglycaemia, which is reported less frequently and variably across studies [3,9]. Other reported indications include marginal ulceration (14.6%), intractable nausea and vomiting, and chronic abdominal pain (10.4%), although the frequency of these indications varies between studies and is not always reported as separate categories [5,8,9]. Reversal is technically complex and associated with substantial postoperative morbidity. In one multicentre cohort, overall morbidity during the first postoperative year reached 50%, with 16.7% classified as major complications (Clavien-Dindo grade III-IV) [9]. Nevertheless, symptom remission has been reported in approximately 50%-100% of patients [3]. Thus, reversal carries notable risks but may provide meaningful quality-of-life benefits in selected cases. To the best of the authors’ knowledge, reports describing isolated, refractory nausea following RYGB in the absence of identifiable structural or biochemical abnormalities remain limited. This highlights a gap in the current literature and illustrates the diagnostic and therapeutic challenges associated with such postoperative symptoms. The following report details the clinical course, management, and outcome of this single patient case.

## Case presentation

**Figure 1:**
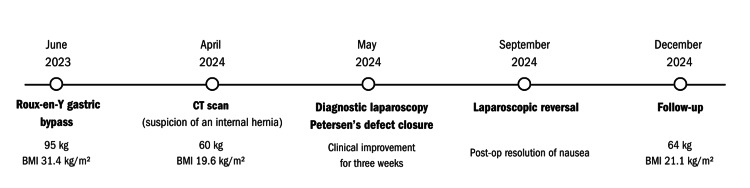
Timeline of the patient’s clinical course The figure illustrates key diagnostic findings, surgical interventions, symptom progression, and weight changes from initial RYGB to postoperative follow-up after reversal. RYGB, Roux-en-Y gastric bypass.

A 39-year-old female presented to a bariatric surgery clinic seeking assistance with weight management and reported long-standing difficulty achieving meaningful weight loss through lifestyle modifications. The patient’s clinical course, including key diagnostic findings, interventions, and follow-up, is summarised in Figure 1. Written informed consent was obtained from the patient for publication of this case report. Before referral, the patient had attempted weight reduction through structured dietary interventions and increased physical activity but was unable to achieve sustained results. She declined pharmacological therapy for weight loss despite it being offered. At evaluation, her weight was 95 kg, corresponding to a body mass index (BMI) of 31.4 kg/m². Her medical history was notable for chronic musculoskeletal pain involving the lower back and ankles and gastroesophageal reflux disease (GERD), confirmed by esophagogastroduodenoscopy showing reflux esophagitis (Los Angeles grade B). Preoperative assessment was conducted by a multidisciplinary bariatric team, including a bariatric surgeon, a nutrition specialist, and a coordinator, who provided detailed information regarding surgical risks, potential benefits, and possible disadvantages, as well as evaluated factors that might contraindicate bariatric surgery. When concerns arise, additional specialists (e.g., psychologist) are involved, or external specialist evaluations are requested if the patient is already under their care. In this case, the patient was deemed an appropriate candidate for bariatric surgery. She met the International Federation for the Surgery of Obesity and Metabolic Disorders (IFSO) criteria for consideration of metabolic and bariatric surgery (BMI: 30.0-34.9 kg/m² with comorbidities) [10]. The decision to proceed with surgery at this BMI was based on the 2022 American Society for Metabolic and Bariatric Surgery (ASMBS)-IFSO guidelines [10], given the presence of obesity-related comorbidities, failure of lifestyle interventions, and the patient’s refusal of pharmacological weight loss therapy, which is generally considered before surgical intervention. RYGB was selected over sleeve gastrectomy because of GERD, as RYGB is associated with a lower risk of GERD exacerbation than sleeve gastrectomy [11].

Her surgical history included laparoscopic bilateral ovarian cystectomy in 2007 and laparoscopic cholecystectomy in 2011. At presentation, medications included ibuprofen 400 mg orally once or twice daily for musculoskeletal pain and pantoprazole 20 mg orally once daily for GERD. She denied tobacco or alcohol use and had no other significant comorbidities.

The patient underwent elective laparoscopic RYGB on June 1, 2023. The operative time was 60 minutes, and the immediate postoperative course was uneventful. She was discharged on postoperative day two and continued regular nutritional counselling.

Five months postoperatively, she developed persistent nausea, anorexia, asthenia, and an inability to gain or maintain body weight. Her weight declined from 95 kg to 60 kg over the subsequent five months, resulting in a BMI of 19.6 kg/m². Conservative management with dietary modifications and antiemetic therapy (metoclopramide 10 mg orally three times daily) failed to relieve symptoms. No additional antiemetic agents, neuromodulators, or gut-brain axis-targeted therapies were trialled, as further pharmacological treatment options were declined by the patient. These could have included alternative antiemetics (e.g., ondansetron), neuromodulators (e.g., tricyclic antidepressants or mirtazapine), or therapies targeting the gut-brain axis, which may be beneficial in functional or centrally mediated gastrointestinal symptoms. This represents a limitation of the conservative management, as a broader and more structured pharmacological approach could have been considered before surgical intervention. Consequently, it remains uncertain whether such therapies might have improved symptom control and potentially allowed avoidance of surgical intervention.

**Figure 2:**
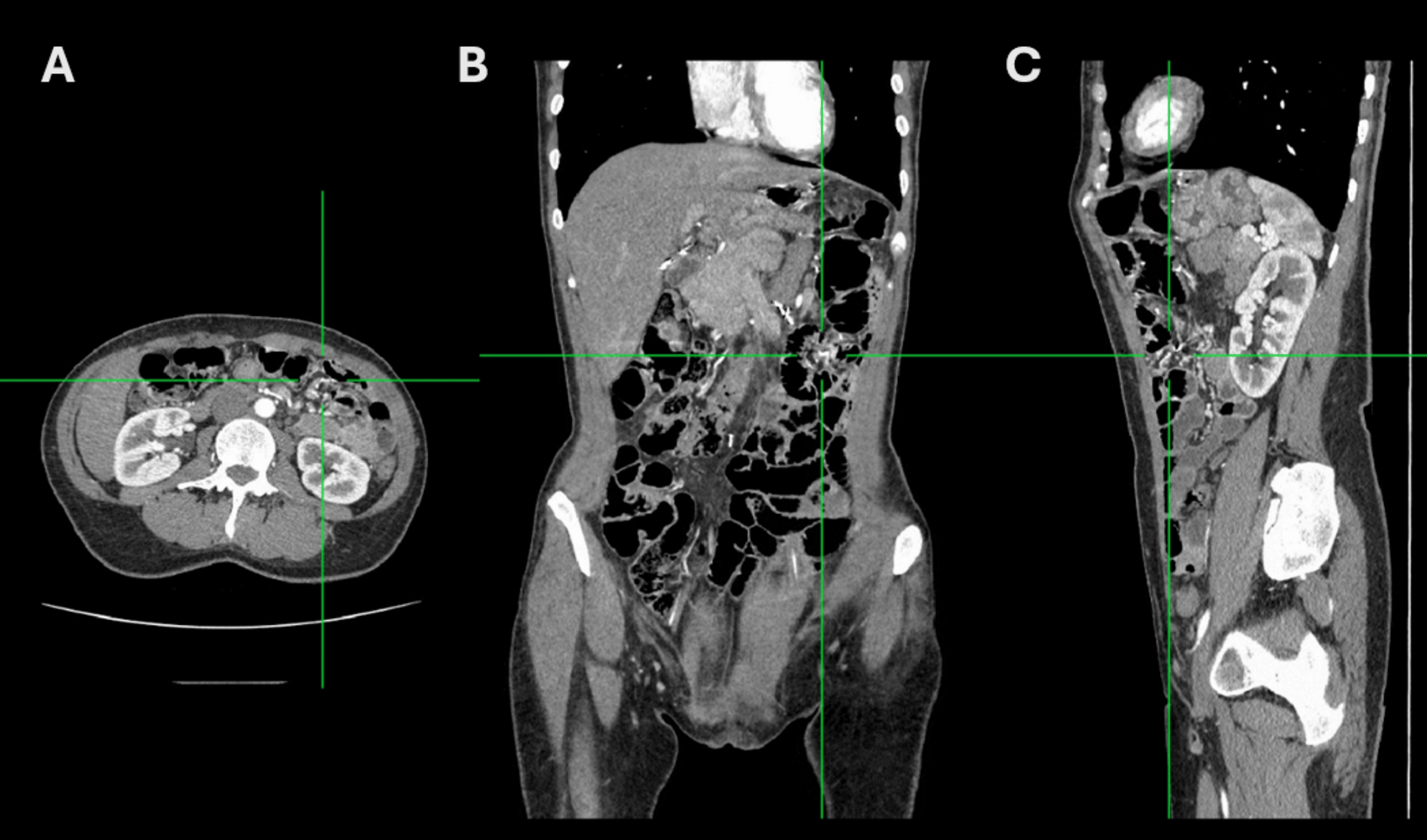
Abdominal CT scan Contrast-enhanced abdominal CT images. Axial (A) and coronal (B) views demonstrate clustering of small bowel loops predominantly in the left abdominal flank. Although no definitive radiological signs of internal herniation, such as the whirlpool sign, are identified, these findings are considered non-specific but clinically suspicious in the context of the patient’s symptoms, thereby contributing to the decision for surgical exploration. Axial (A), coronal (B), and sagittal (C) views additionally show a mildly dilated hepatobiliary limb containing fluid, most consistent with bile.

**Figure 3:**
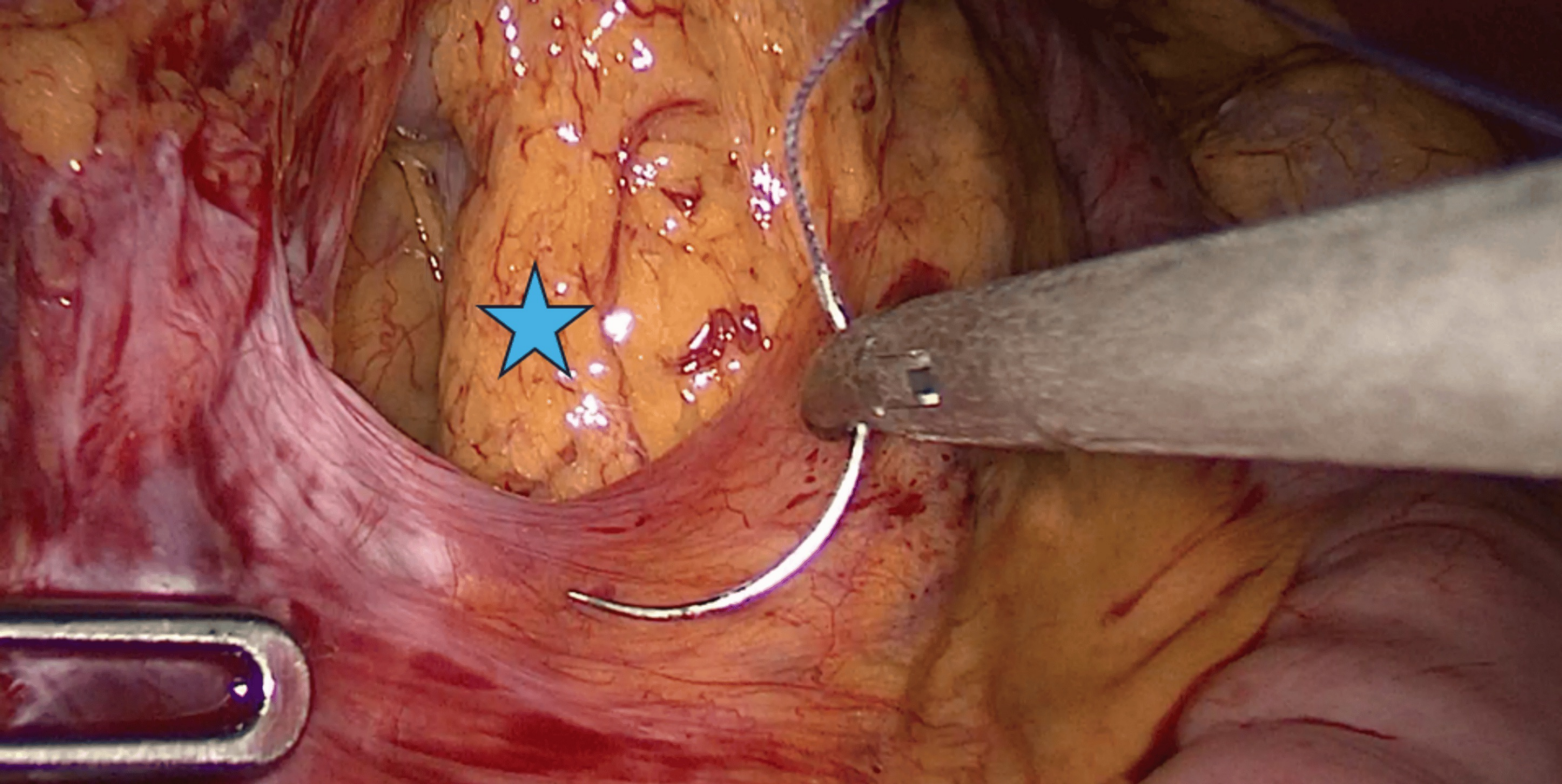
Petersen’s defect Intraoperative view demonstrating Petersen’s defect (blue star) identified during diagnostic laparoscopy. The defect is located between the mesentery of the alimentary limb and the transverse mesocolon and represents a potential site for internal herniation following RYGB. The defect was closed using 3-0 braided absorbable sutures to reduce the risk of future internal herniation.

A contrast-enhanced CT scan performed on April 9, 2024, demonstrated clustering of small bowel loops in the left abdominal quadrant, raising suspicion of an internal hernia; however, no whirlpool sign was observed (Figure 2). The patient was readmitted on May 2, 2024, approximately 11 months after the primary RYGB. Diagnostic laparoscopy identified Petersen’s defect, which was closed using braided absorbable sutures (Figure 3). The defect was closed to prevent the development of a future internal hernia. No internal hernia was observed, and no other intra-abdominal abnormalities were identified. The procedure lasted 30 minutes, and the patient was discharged on postoperative day one. Postoperative laboratory results were within normal limits, including complete blood count, coagulation profile, serum iron and ferritin, liver and renal function parameters, total protein, glucose, sodium, potassium, lipid profile, and thyroid-stimulating hormone.

Following closure of Petersen’s defect, the patient experienced transient clinical improvement lasting three weeks, after which nausea, anorexia, asthenia, and an inability to maintain body weight recurred. Given the persistence and severity of her symptoms despite unremarkable radiologic or biochemical abnormalities, together with a marked deterioration in quality of life and the patient’s expressed preferences, further conservative pharmacologic therapy was considered unlikely to be beneficial. The patient declined additional conservative treatment options, including psychological or psychiatric evaluation, antidepressant therapy, and other pharmacological approaches for symptom control. Furthermore, no structured evaluation for functional gastrointestinal disorders, somatic symptom disorders, or eating-related behavioural issues was performed. Although initial preoperative assessment before the primary RYGB included evaluation by a bariatric surgeon, coordinator, and a nutrition specialist, this does not replace a formal, structured psychological or functional assessment. This represents an additional limitation of the diagnostic and conservative management approach. Given the persistence and severity of symptoms, together with the patient’s preferences and limited remaining non-surgical options, the multidisciplinary team decided to proceed with laparoscopic reversal of the RYGB to normal anatomy.

**Figure 4:**
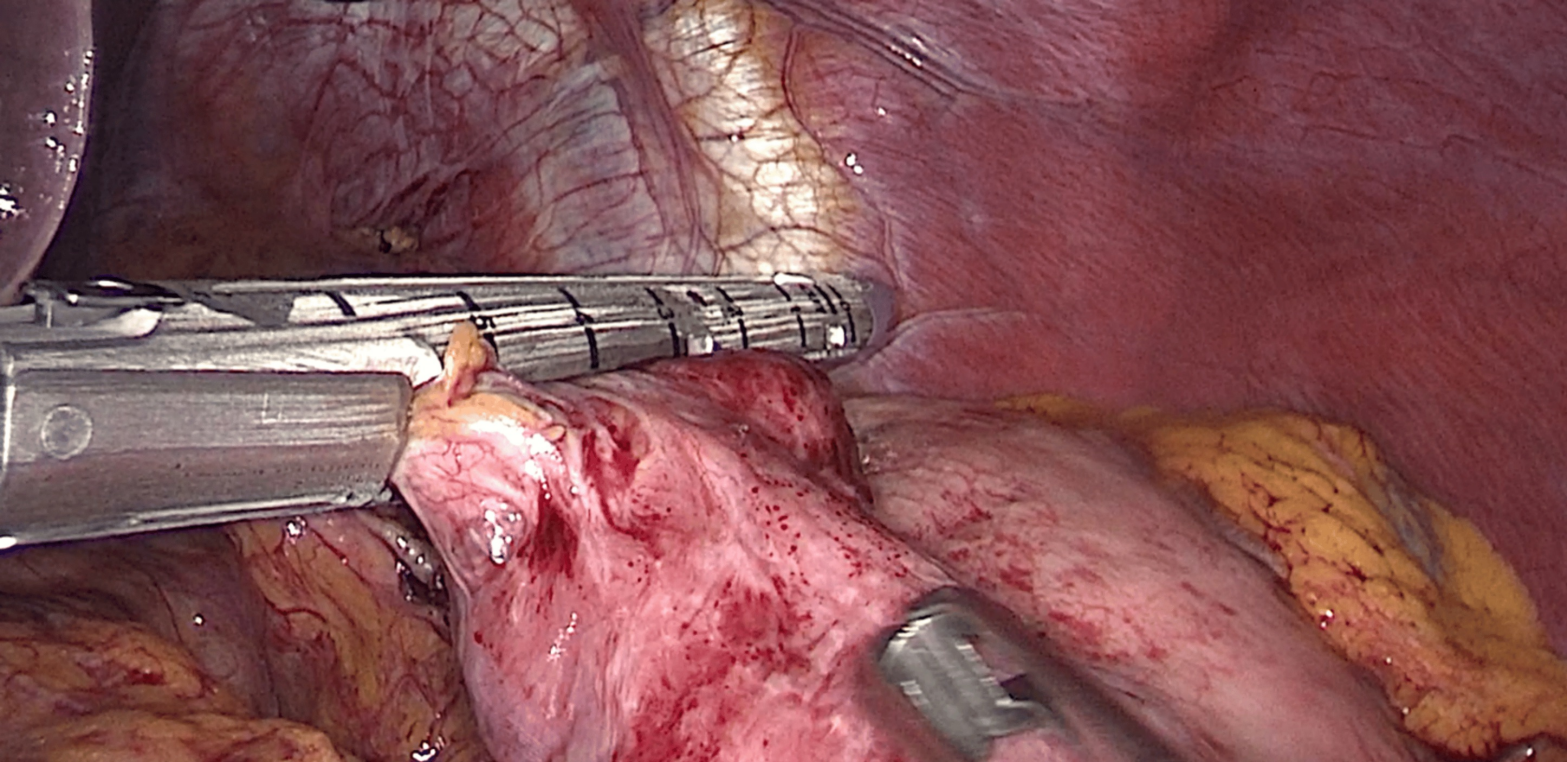
Transection of gastrojejunal anastomosis Intraoperative image showing transection of the gastric pouch proximal to the gastrojejunal anastomosis using a 65 mm violet cartridge endoscopic linear stapler as part of reversal to normal anatomy.

**Figure 5:**
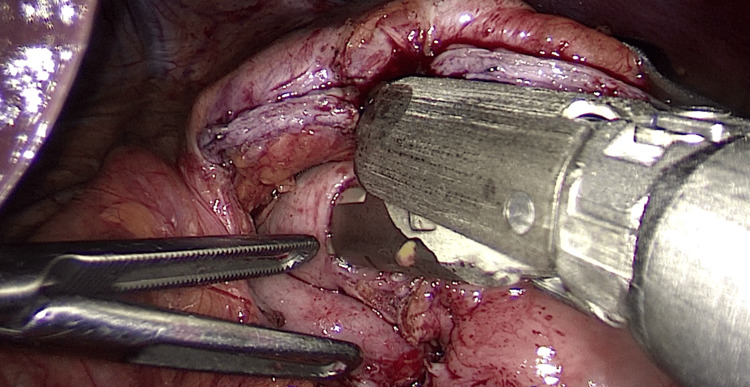
Creation of a side-to-side anastomosis Intraoperative image demonstrating creation of a stapled side-to-side gastrogastric anastomosis between the gastric pouch (top) and remnant stomach (bottom) using a 45 mm gold cartridge endoscopic linear stapler. The anastomotic defect was subsequently closed in two layers with a continuous 3-0 braided absorbable suture, restoring continuity of the stomach.

**Figure 6:**
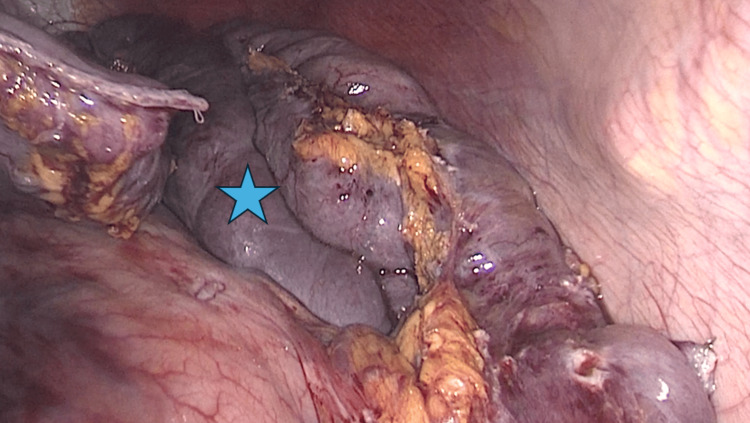
Resected alimentary limb Intraoperative image showing the resected 1.5 m of alimentary Roux limb (blue star), which was mobilised and divided up to the level of the enteroenteric anastomosis. The specimen was retrieved using a surgical extraction bag.

On September 15, 2024, the patient reported loose stools, a sensation of heaviness after meals, fatigue, lightheadedness, night sweats, abdominal bloating, poor sleep, and difficulty maintaining body weight. Her weight at that time was 61 kg. Preoperative upper gastrointestinal endoscopy performed before reversal demonstrated no significant abnormalities apart from signs of cardia insufficiency. On September 18, 2024, 15 months after the initial RYGB and four months after closure of Petersen’s defect, she underwent laparoscopic conversion to normal anatomy. Using a harmonic scalpel, the small gastric pouch was mobilised and transected above the anastomosis with an endoscopic linear stapler, using a 65 mm violet cartridge (Figure 4). Approximately 5 cm below the oesophagus, the small curvature of the stomach was dissected towards the posterior gastric wall, which was further mobilised using the harmonic scalpel. A side-to-side anastomosis was created with an endoscopic linear cartridge, using a 45 mm gold cartridge (Figure 5). The anastomotic defect was closed in two layers with a continuous 3-0 braided absorbable suture. The alimentary limb was resected up to the enteroenteric anastomosis and retrieved, with the resected Roux limb measuring approximately 1.5 m (Figure 6). Haemostasis along the staple line was achieved using laparoscopic clips. At the end of the procedure, the integrity of the anastomosis was assessed using a methylene blue test, with diluted methylene blue solution (in 0.9% NaCl) administered into the stomach via an orogastric tube. The procedure was completed in 85 minutes without complications.

At follow-up on December 18, 2024, four months after the reversal, the patient remained free of severe symptoms, including nausea, anorexia, and weakness. Her weight had increased to 64 kg (BMI: 21.1 kg/m²). She also reported resolution of chronic low back and ankle pain and complete remission of GERD symptoms, with discontinuation of daily non-steroidal anti-inflammatory drugs and proton pump inhibitors as a result of weight loss. Postoperatively, the patient was advised to continue outpatient follow-up with a surgeon, avoid heavy physical activity for four weeks, adhere to a semi-liquid diet for three weeks, undergo laboratory testing at 1, 3, 6, and 12 months, and remain under the supervision of a dietitian.

The patient was contacted via e-mail in December 2025. The patient did not attend scheduled in-person follow-up visits by her own preference; therefore, subsequent follow-up was conducted remotely. She reported no ongoing symptoms, good overall health, and adequate weight control achieved through physical activity and a diet high in protein, and that her laboratory test results were within normal limits. She declined to provide any additional information and indicated she did not wish to engage in further discussion on this matter. This decision was attributed to a reluctance to revisit a psychologically distressing period, which she described as particularly difficult. Nevertheless, she expressed gratitude to the medical team for the care provided.

## Discussion

Although early postoperative nausea is common and well studied [12], persistent and treatment-resistant nausea after bariatric surgery remains poorly understood. There is no consensus regarding the timing or indications for reversal surgery in such cases [9]. Most studies report the number of patients undergoing reversal for specific indications, but none have focused exclusively on refractory nausea as a complication after bariatric surgery. To our knowledge, reports describing refractory nausea as the primary indication for RYGB reversal remain limited, and this case contributes to the understanding of this rare and poorly characterised clinical scenario. This case report provides additional clinical insight into an underexplored complication and highlights refractory nausea as a rare and poorly characterised potential indication for reversal surgery in selected patients. Importantly, it also illustrates the diagnostic and therapeutic challenges in distinguishing between functional, psychological, and organic causes of persistent postoperative symptoms. The patient’s clinical course is summarised in Figure 1.

RYGB reversal is associated with substantial risk. Reported complications include anastomotic leak, gastric ulceration, persistent abdominal pain, portal vein thrombosis, and septicaemia [8]. Given these risks, non-surgical management, including pharmacologic therapy, nutritional optimisation, and psychological support, should be exhausted before considering reversal. In this case, the patient received nutritional counselling and treatment with a single antiemetic agent, which did not provide symptom relief. A key limitation is that only one pharmacologic agent was trialled, despite the availability of alternative therapies, including prokinetics, neuromodulators, and certain antidepressants that may be beneficial in functional or centrally mediated gastrointestinal symptoms [13]. Furthermore, the patient declined psychological or psychiatric evaluation and additional pharmacological treatment, which further limited conservative management options. Consequently, it remains uncertain whether a more comprehensive non-surgical approach could have improved her condition. In the absence of well-defined clinical guidelines, the threshold for transitioning from conservative therapy to surgical intervention remains unclear. Additionally, structured clinical follow-up was limited to four months, with subsequent information obtained via remote communication, which may limit the reliability of long-term outcome assessment.

This case also raises important considerations regarding clinical decision-making. Although the patient’s BMI was at the lower end of the surgical eligibility range, the presence of obesity-related comorbidities, failure of lifestyle interventions, and refusal of pharmacological therapy supported the decision for surgical management in accordance with the current 2022 ASMBS-IFSO guidelines [10]. It remains uncertain whether conservative management was sufficiently exhaustive before reversal, as only a limited range of pharmacological therapies was trialled, and a psychological assessment was not performed. In addition, the possibility of an underlying functional gastrointestinal disorder cannot be excluded, given the absence of clear structural abnormalities on imaging and endoscopy. Functional nausea is thought to arise from dysregulation of the gut-brain axis, involving altered central processing of visceral signals, autonomic dysfunction, and increased visceral sensitivity. These conditions fall within the spectrum of disorders of gut-brain interaction, which are characterised by chronic gastrointestinal symptoms in the absence of structural pathology and may include abdominal pain, nausea, bloating, constipation, and diarrhoea. They are frequently associated with psychological and behavioural factors [14]. Management of these conditions typically involves a multimodal, conservative approach, including pharmacological therapies such as antiemetics, prokinetics, and neuromodulators (e.g., tricyclic antidepressants or mirtazapine), as well as psychological interventions targeting the gut-brain axis [14]. Although standard diagnostic investigations were performed, a more specialised evaluation for functional gastrointestinal disorders was not undertaken, and additional assessments such as gastric emptying studies, gastrointestinal motility testing, or evaluation for small intestinal bacterial overgrowth (SIBO) were not performed, which represents a limitation in the diagnostic workup. Furthermore, such structured and comprehensive conservative management was not fully implemented, as the patient declined additional pharmacological and psychological treatment options. Therefore, it remains uncertain whether these approaches might have improved symptom control and potentially avoided the need for surgical intervention.

Ma et al. suggested that psychological factors might play a role in postoperative symptoms [5]. They proposed that some patients may not have been psychologically prepared for the lifestyle changes required after bariatric surgery, potentially resulting in persistent symptoms such as nausea or abdominal pain without an identifiable organic cause. In the present case, a psychological contribution cannot be excluded; however, no formal psychiatric or psychological assessment was performed, as the patient declined such evaluation. Therefore, any interpretation regarding psychological or psychosomatic factors remains speculative. The patient later described the pre-reversal period as psychologically distressing, further suggesting that non-organic factors may have contributed to her clinical presentation. This highlights the potential value of more rigorous preoperative psychological screening in bariatric surgery candidates. 

Risk factors also warrant attention. Current evidence on risk factors for postoperative nausea and vomiting primarily relates to the early postoperative period, and there are no well-established predictors of persistent nausea several months after bariatric surgery. Further research is needed to determine whether known early risk factors, such as female sex, longer operative time, greater blood loss, and intraoperative hypotension [15], are associated with prolonged postoperative nausea. Additionally, patient-related factors such as psychological vulnerability, treatment adherence, and refusal of recommended therapies may also play a role and warrant further investigation. Identification of such predictors could support more targeted prevention and follow-up strategies.

Based on this case, several practical considerations may be proposed. In patients presenting with persistent, treatment-resistant nausea after bariatric surgery, a stepwise approach should be considered: (1) exclusion of surgical complications (e.g., internal hernia, obstruction), as well as consideration of additional functional investigations such as gastric emptying studies, gastrointestinal motility assessment, and evaluation for SIBO, (2) comprehensive nutritional and metabolic assessment, including evaluation for micronutrient deficiencies and postprandial hypoglycaemia, (3) trial of multiple pharmacological therapies, including antiemetics, prokinetics, and neuromodulators, (4) formal psychological or psychiatric evaluation when symptoms remain unexplained, and (5) multidisciplinary team discussion before considering surgical reversal. Reversal surgery should be reserved for carefully selected patients with severe, persistent symptoms that significantly impair quality of life and remain refractory to comprehensive conservative management.

An additional consideration is the healthcare system context in which this case was managed. Although international guidelines, such as the 2022 ASMBS-IFSO recommendations [10], are available, there are currently no national guidelines governing the management of bariatric patients in this setting. As a result, clinical decision-making is largely individualised and dependent on multidisciplinary team judgment. Furthermore, access to recommended investigations and specialist consultations may be limited by the patient’s financial resources, as publicly funded services for comprehensive obesity management are not available. These factors may influence both diagnostic evaluation and treatment pathways in real-world clinical practice.

Until clinical guidelines define more precise diagnostic pathways and management strategies for persistent, therapy-resistant symptoms following bariatric surgery, treatment decisions must rely on individual clinical judgment and available evidence. Conservative approaches should be maximised whenever feasible to reduce exposure to the risks of reoperation. However, patient-related factors, including refusal of recommended nonsurgical therapies, must also be considered within a shared decision-making framework. In selected cases, reversal surgery may represent a reasonable option when severe symptoms persist and significantly impair quality of life despite limited therapeutic alternatives.

## Conclusions

Although bariatric surgery is effective in the management of obesity, rare but severe complications may occur. This case demonstrates that persistent, therapy-resistant nausea and anorexia can develop after RYGB even in the absence of clear radiologic or biochemical abnormalities, leading to significant weight loss and functional impairment. To the best of the authors’ knowledge, reports describing refractory nausea as the primary indication for RYGB reversal remain limited. This case contributes to the existing literature by highlighting a rare clinical scenario and the associated diagnostic and management challenges. However, the findings of this report should be interpreted with caution, given the limitations of incomplete diagnostic workup, restricted conservative management, and limited structured follow-up.

This case underscores that comprehensive multidisciplinary evaluation is mandatory in patients with unexplained postoperative symptoms and should include surgical, nutritional, pharmacological, and, where feasible, psychological or psychiatric assessment. Exhaustive diagnostic workup and non-surgical management must be prioritised before considering operative intervention. Reversal to normal anatomy should be regarded strictly as a last-resort option, reserved for carefully selected patients with severe, persistent symptoms that significantly impair quality of life and remain refractory to all available conservative measures. Clinical decision-making in such scenarios must balance surgical risk against symptom burden and incorporate patient preferences within a structured multidisciplinary framework. Further studies are needed to clarify diagnostic approaches, identify potential predictive factors, and establish evidence-based recommendations for the management of persistent, refractory symptoms following bariatric surgery.
